# Rare anatomical variation of the inferior turbinate: two cases of concha bullosa with review of the literature

**DOI:** 10.1093/jscr/rjaf979

**Published:** 2025-12-12

**Authors:** Abulrahman Subaih, Batool Alzahir, Aljohara Almazroua, Abdulrahman Al-Harbi, Ali Almomen

**Affiliations:** Royal Commission Hospital, Royal Commission for Jubail and Yanbu, Jubail 31961, Saudi Arabia; Department of Otolaryngology Head and Neck Surgery, King Fahad Specialist Hospital, Dammam 32253, Saudi Arabia; Department of Otolaryngology Head and Neck Surgery, King Fahad Specialist Hospital, Dammam 32253, Saudi Arabia; Department of Rhinology & Skull Base Surgery, King Fahad Specialist Hospital, Dammam 32253, Saudi Arabia; Department of Rhinology & Skull Base Surgery, King Fahad Specialist Hospital, Dammam 32253, Saudi Arabia

**Keywords:** inferior concha bullosa, nasal turbinates, concha bullosa, endoscopic sinus surgery, anatomical variation, CT imaging

## Abstract

Inferior concha bullosa (ICB) is a rare anatomical variation characterized by pneumatization of the inferior nasal turbinate. It is usually asymptomatic and discovered incidentally on computed tomography (CT) imaging but may contribute to nasal obstruction or rhinosinusitis in certain patients. We present two cases of ICB diagnosed on CT imaging. One patient underwent endoscopic sinus surgery with resolution of symptoms, while the second was managed successfully with conservative medical therapy. These cases highlights the importance of considering ICB in the differential diagnosis of nasal obstruction and emphasizes CT imaging as the diagnostic modality of choice.

## Introduction

Intranasal turbinates are bony projections that extend from the lateral walls of the nasal cavity. Anatomically, the superior and middle turbinates are extensions of the ethmoid bone, whereas the inferior turbinate is a separate, independent bony structure. These turbinates are susceptible to various pathophysiological processes, including inflammatory changes secondary to infection or hypersensitivity reactions following allergen exposure. Such conditions may result in turbinate hypertrophy and nasal obstruction, thereby impairing ventilation of the osteomeatal complex and inhibiting mucociliary drainage from the paranasal sinuses—both of which are important factors in the development and persistence of chronic rhinosinusitis.

Concha bullosa (CB) refers to the pneumatization of a nasal turbinate and is considered the most common anatomical variation within the sinonasal region. This condition predominantly affects the middle turbinate and is rarely observed in the superior or inferior turbinates [1]. Several studies have indicated that inferior concha bullosa (ICB) is the least common form of turbinate pneumatization. Although CB is often asymptomatic and discovered incidentally on computed tomography (CT) imaging, it may require clinical attention when associated with nasal obstruction or when it contributes to sinonasal pathology.

## Case 1

A 35-year-old male with no significant medical history presented to the rhinology clinic with persistent nasal obstruction and intermittent headaches over the past several months. He denied facial pressure, olfactory disturbances, or postnasal drip. There was no history of prior nasal or sinus surgery.

**Figure 1:**
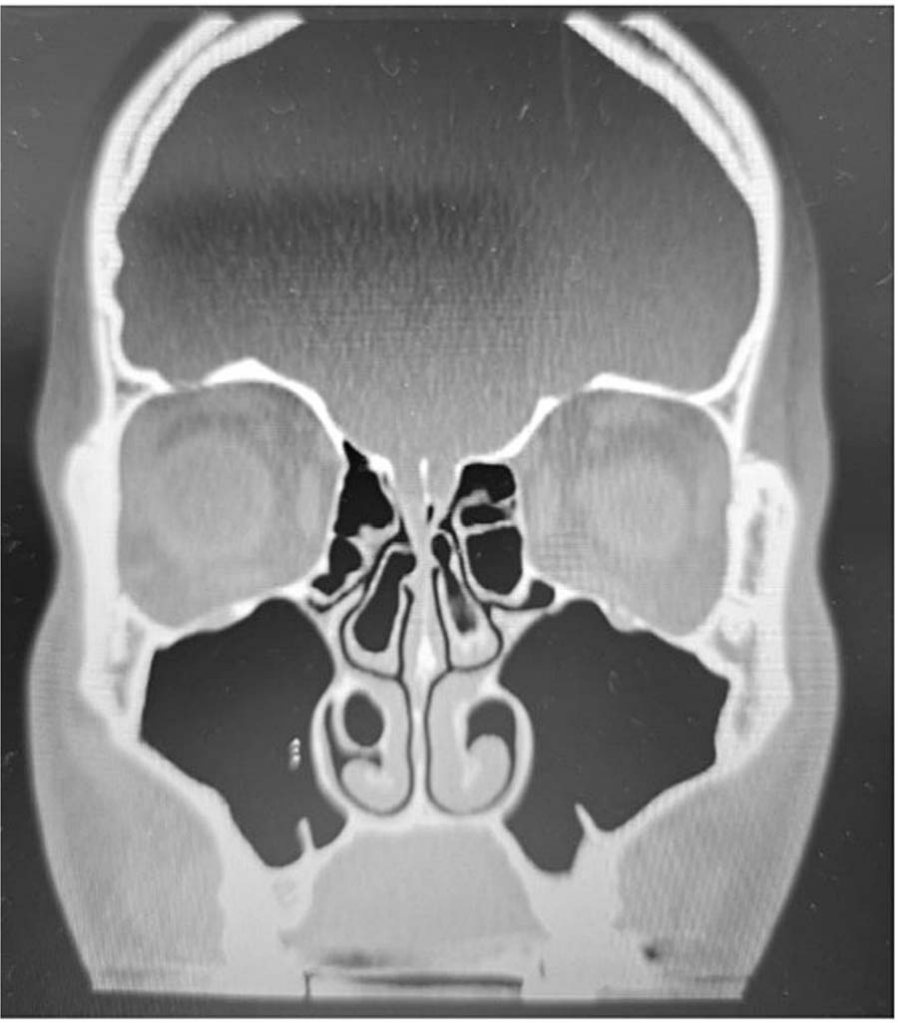
Paranasal sinus CT scan in the coronal plane showing bilateral concha bullosa in the superior turbinates and pneumatization of the right inferior turbinate (inferior concha bullosa).

**Figure 2:**
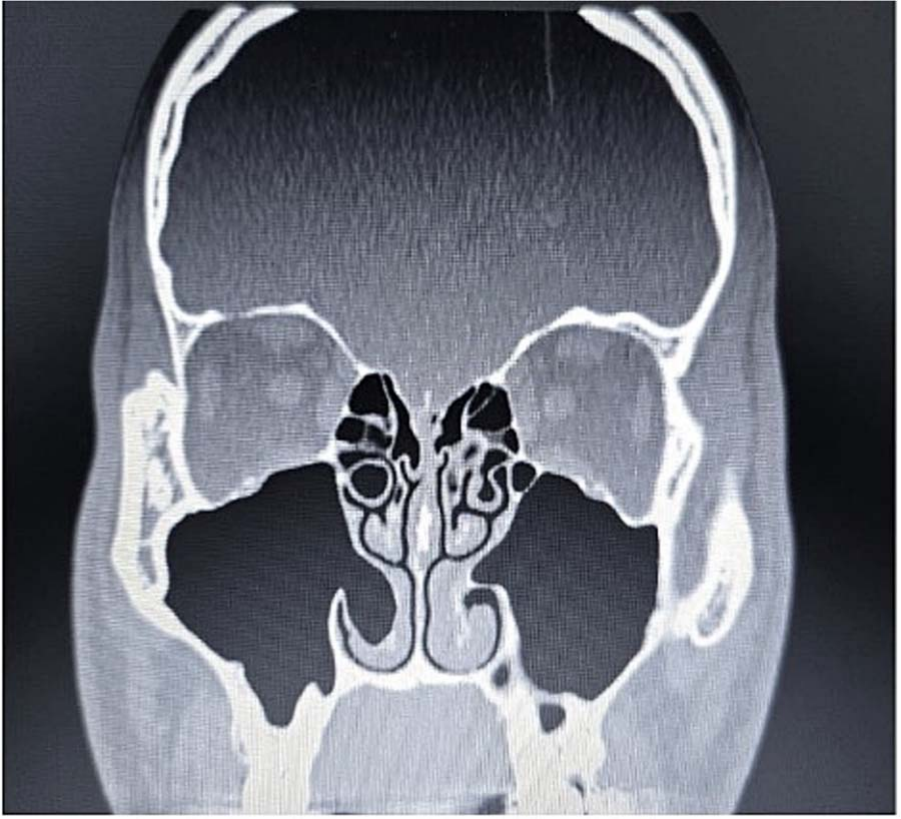
Paranasal sinus CT scan in the coronal plane demonstrating bilateral inferior concha bullosa with communication to the maxillary sinuses.

Clinical examination, including anterior rhinoscopy and nasal endoscopy, revealed a midline nasal septum and bilateral hypertrophy of the inferior and middle turbinates, with otherwise normal nasal mucosa. A CT scan of the paranasal sinuses in the coronal plane demonstrated bilateral pneumatization of the middle turbinates consistent with CB. Additionally, the right inferior turbinate was pneumatized, with communication to the ipsilateral maxillary sinus (Figs 1 and 2).

Following thorough counseling regarding surgical options and potential outcomes, the patient underwent endoscopic sinus surgery. The procedure included a middle meatus antrostomy and bilateral endoscopic turbinoplasty, targeting the inferior and posterior third of the inferior turbinate to reduce turbinate bulk. The patient was discharged on the same day. At postoperative follow-up, he reported significant symptomatic improvement with no residual complaints.

## Case 2

A 35-year-old medically healthy, nonsmoking male presented to the rhinology clinic with a 6-month history of alternating bilateral nasal obstruction. He denied headaches, facial pressure, or changes in smell.

**Figure 3:**
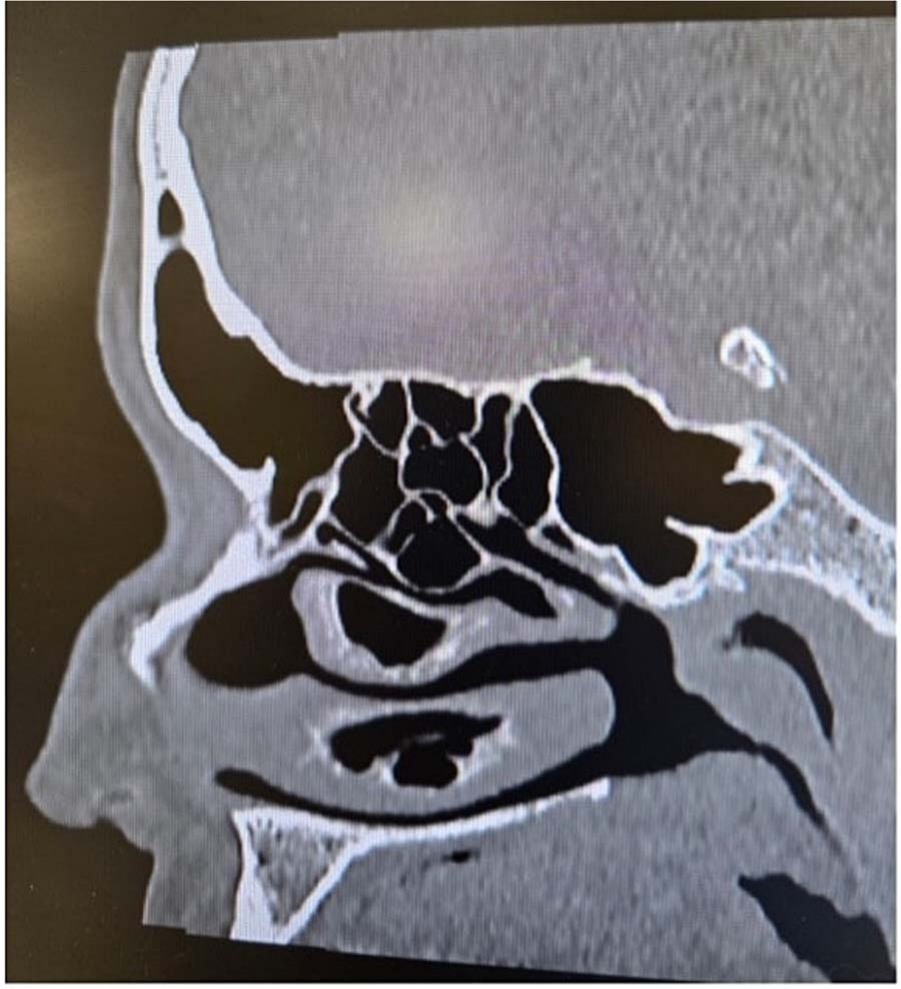
Paranasal sinus CT scan in the sagittal plane demonstrating middle concha bullosa and inferior concha bullosa.

**Figure 4:**
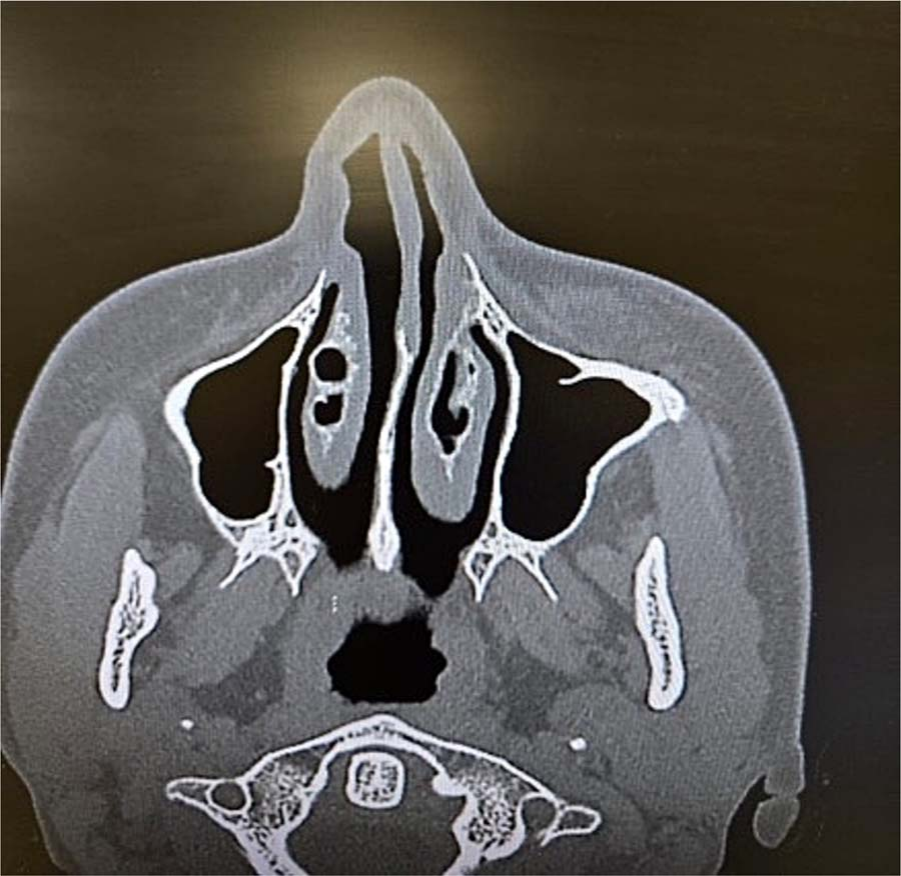
Paranasal sinus CT scan in the axial plane demonstrating inferior concha bullosa.

**Figure 5:**
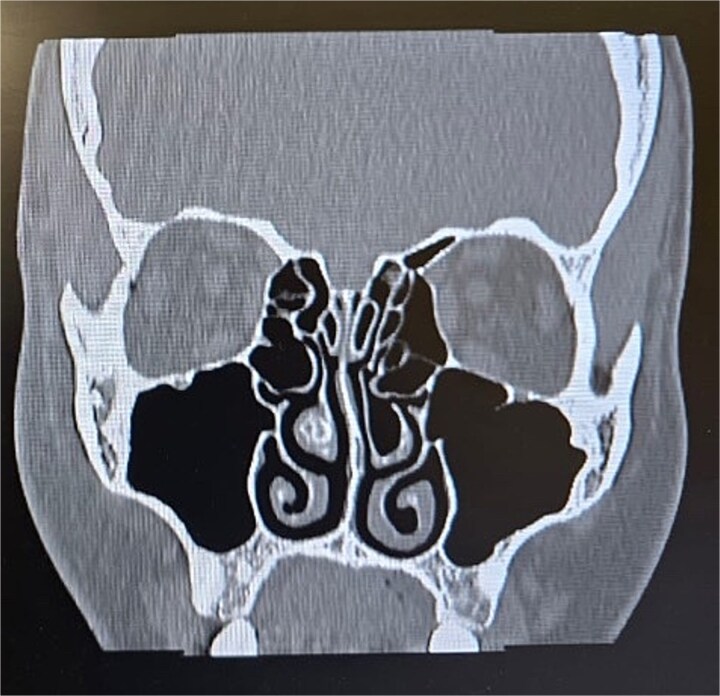
Paranasal sinus CT scan in the coronal plane demonstrating left middle concha bullosa and inferior concha bullosa.

Anterior rhinoscopy and nasal endoscopy demonstrated bilateral hypertrophy of the inferior and middle turbinates, with clear and patent sinus ostia and no evidence of polyps or masses. A coronal CT scan of the paranasal sinuses revealed bilateral ICB and a unilateral CB of the left middle turbinate (Figs 3–5).

The patient was treated conservatively with a topical intranasal corticosteroid. Follow-up was scheduled six months after initiating medical therapy, at which time the patient reported notable improvement in nasal symptoms.

## Discussion

Intranasal turbinates, or conchae, are critical anatomical structures of the nasal cavity, projecting from the lateral nasal wall and playing a fundamental role in nasal physiology. Typically, there are three paired turbinates: superior, middle, and inferior. The superior and middle turbinates arise from the ethmoid bone, while the inferior turbinate is a separate bony structure. In some anatomical variations, a supreme turbinate may also be identified, originating from the ethmoid bone.

During the 8th to 10th weeks of fetal development, the nasal turbinates begin to form from embryologic precursors: the ethmoturbinal and maxilloturbinal. The ethmoturbinal gives rise to the superior, middle, and occasionally the supreme turbinates, whereas the maxilloturbinal forms the inferior turbinate [1]. These structures are lined with highly vascularized mucosa composed of pseudostratified ciliated columnar epithelium containing numerous goblet cells [2]. The turbinates contribute significantly to the regulation of inspired air temperature, humidification, and filtration, serving as a first-line defense against airborne pathogens and environmental irritants.

CB refers to the pneumatization of a nasal turbinate and most commonly involves the middle turbinate, followed by the superior turbinate, with rare occurrence in the inferior turbinate. A retrospective study conducted at Creighton University reported a CB prevalence of 67.5% in 883 cone-beam CT scans, although the specific turbinates involved were not delineated [3]. Another radiological study conducted at Busan Saint Mary’s Hospital involving 200 sinusitis patients found unilateral and bilateral middle turbinate CB in 17.3% and 36.4% of cases, respectively. Superior turbinate CB occurred unilaterally in 11.3% and bilaterally in 27.4%. ICB was identified in only 1% of patients [4]. Similarly, Kar *et al*. reported ICB in only 1 of 3133 patients (0.0003%), underscoring the rarity of this anatomical variant [5].

ICB is typically asymptomatic and discovered incidentally on CT imaging. However, it may present with symptoms consistent with chronic rhinosinusitis, including nasal obstruction, headache, nasal discharge, anosmia, or hyposmia [6–8]. These symptoms are generally attributed to obstruction of the osteomeatal complex caused by enlarged or pneumatized turbinates, which disrupt normal sinus drainage.

CT imaging is the modality of choice for evaluating turbinate pneumatization. On CT, CB appears as a low-attenuation cavity surrounded by thin bony walls. In asymptomatic patients, no intervention is typically required. However, symptomatic cases may necessitate treatment depending on the severity and impact on nasal airflow.

Initial management is usually conservative and includes intranasal corticosteroids and antihistamines, especially in patients presenting with obstructive symptoms. Surgical intervention, most often in the form of functional endoscopic sinus surgery, is reserved for patients who do not respond to medical therapy or those with radiographic evidence of extensive sinonasal disease [7–9].

## Conclusion

ICB is a rare anatomical variation of the lateral nasal wall, most often asymptomatic and incidentally detected on CT imaging. However, in select cases, it may contribute to clinically significant symptoms, including nasal obstruction and recurrent rhinosinusitis. This report presents two cases of ICB, one managed conservatively and the other surgically, both demonstrating favorable outcomes. These findings underscore the importance of considering ICB in the differential diagnosis of sinonasal symptoms and highlight the role of individualized management based on symptom severity and radiologic findings.
